# On the use of single‐isocenter VMAT plans for SBRT treatment of synchronous multiple lung lesions: Plan quality, treatment efficiency, and early clinical outcomes

**DOI:** 10.1002/acm2.12938

**Published:** 2020-06-17

**Authors:** Damodar Pokhrel, Lana Sanford, Shilpa Larkin, Bhaswanth Dhanireddy, Mark E. Bernard, Marcus Randall, Ronald C. McGarry

**Affiliations:** ^1^ Department of Radiation Medicine Medical Physics Graduate Program University of Kentucky Lexington KY USA

**Keywords:** clinical outcomes, FFF beams, lung SBRT, oligometastastic lesions, single‐isocenter VMAT

## Abstract

Cone‐beam computed tomography (CT)‐guided volumetric‐modulated arc therapy (VMAT) plans for stereotactic body radiotherapy (SBRT) treatment of synchronous multiple lung lesions with a flattening filter‐free (FFF) beam is a safe and highly effective treatment option for oligometastases lung cancer patients. Fourteen patients with metastatic non–small‐cell lung cancer (NSCLC) lesions (two to five) received a single‐isocenter VMAT SBRT treatment in our clinic. Four‐dimensional (4D) CT‐based treatment plans were generated using advanced AcurosXB‐based dose calculation algorithm using heterogeneity corrections with a single isocenter placed between/among the lesions. Compared to 10X‐FFF and traditional flattened 6X (6X‐FF) beams, 6X‐FFF beam produced highly conformal radiosurgical dose distribution to each target volume, reduced dose to adjacent organs at risk (OAR), and significantly reduced the lung SBRT fraction duration to < 3.5 min/fraction for 54/50 Gy treatments in 3/5 fractions — significantly improving patient convenience and clinic workflow. Early follow‐up CT imaging (mean, 9 months) results show high local control rates (100%) with no acute lung or rib toxicity. Longer clinical follow up in a larger patient cohort is ongoing to further validate the outcomes of this treatment approach.

## 1 INTRODUCTION

With recent advances in radiotherapy technology, stereotactic body radiotherapy (SBRT) treatment has become a standard curative intent treatment for medically inoperable non–small‐cell lung cancer (NSCLC) patients.^1^, ^2^, ^3^, ^4^, ^5^, ^6^, ^7^ However, some patients with multiple primary or oligometastastic (<5) lung lesions with comorbid illnesses are unable to maintain treatment position adequately for the duration of a SBRT treatment in which individual isocenters are used for each lesion. Furthermore, reducing treatment time would be advantageous in diminishing intrafraction motion errors that accompany longer treatments.^8^, ^9^ Compared to a traditional flattened beam with a flattening filter (FF), flattening filter‐free (FFF) beams have certain advantages, including higher dose rates, reduced lateral beam hardening, and reduced leakage and out‐of‐field dose due to less lateral scattering and electron contamination, without increasing normal tissue toxicity.^10^, ^11^, ^12^ Utilizing FFF‐volumetric modulated arc therapy (VMAT) in the treatment of solitary lung lesions^13^, ^14^, ^15^ and multiple lung lesions synchronously using a single‐isocenter VMAT‐SBRT plan is a fast and efficient treatment technique that is gaining popularity.^16^, ^17^, ^18^

As part of our SBRT commissioning for treating multiple lung lesions synchronously via a single‐isocenter VMAT plan, we have investigated plan quality and treatment efficiency for different beams and reported early clinical outcomes. The dosimetric differences of traditional flattened 6X‐beam (6X‐FF) vs FFF beams for a single‐lesion lung SBRT treatment have been studied previously, along with the feasibility of treating multiple lung lesions concurrently using a single‐isocenter approach.^19^, ^20^, ^21^, ^22^, ^23^, ^24^, ^25^, ^26^ However, plan quality evaluation of FFF beams in the synchronous treatment of multiple lung lesions using a single‐isocenter VMAT‐SBRT plan and the phenomenon of MLC modulation have not yet been investigated. For instance, in a single‐isocenter multifocal VMAT lung SBRT setting, adequate tumor coverage of each lesion requires the MLC leaves to travel a longer distance between the lesions. Moreover, due to considerably softer energy spectra of 6X‐FFF beam (1.28 MeV) compared to conventional 6X‐FF (1.75 MeV), the range of secondary electrons generated by the 6X‐FFF beam in the lung will be shorter and potentially provide quicker dose build up at the lung tissue/tumor interface.^27^

Previous studies of FFF beams focused primarily on improved treatment efficiency when treating a single lung lesion with an isocenter placed at the tumor center. Those patients who developed multiple lung lesions may not tolerate long treatment times associated with multilesion lung SBRT using individual isocenters for each lesion. Additionally, the change in plan quality attributed to the characteristics of FFF beams while treating multiple lung lesions synchronously using a single‐isocenter VMAT plan remains an interesting topic in need of further research, specifically the effect of SBRT on the higher radiosensitivity of nontarget OAR dose.^28^ Therefore, this work was undertaken to quantify the impact of 6X‐FFF beam implementation in the context of single‐isocenter/multilesion VMAT lung SBRT treatment in our clinic and report early clinical findings.

## 2 MATERIALS AND METHODS

### 2.A Patient characteristics and volume delineation

This institutional review board approved retrospective study that includes 14 SBRT patients with 2–5 synchronous metastatic non–small‐cell lung lesions. The patients were immobilized using the Body Pro‐Lok^TM^ platform (CIVCO system, Orange City, IA) in the supine position, arms above their head with abdominal compression. All planning computed tomography (CT) images were acquired on a GE Lightspeed 16 slice CT scanner (General Electric Medical Systems, Waukesha, WI). CT images were acquired with 512 × 512 pixels at 2.5 mm slice thickness. All patients underwent a free breathing scan followed by all 10 phases of a 4D‐CT scan using Varian’s RPM System (version 1.7). Internal target volumes (ITVs) were delineated on the 3D CT images, referenced to the maximum intensity projection (MIP) images, and the planning target volumes (PTVs) were created by adding a 5 mm uniform margin around the corresponding ITVs. Mean combined PTV derived from 4D‐CT scan was 38.7 ± 22.7 cc. The critical structures, such as bilateral lungs excluding the ITV (normal lung), spinal cord, ribs, heart, trachea and bronchus, esophagus, and skin, were delineated on the free‐breathing CT images in the Eclipse treatment planning system (TPS). The main tumor characteristics of the patients are shown in Table 1.

**Table 1:** Main tumor characteristics of the patients included in this study. SD = standard deviation.

Parameters	Mean ± SD (range or no. of patients)
Combined PTV (cc)	38.7 ± 22.7 (15.9–91.8)
Prescription dose (each lesion)	54 Gy in 3 fractions (7 patients)
	50 Gy in 5 fractions (7 patients)
Normal lung volume (cc)	3881 ± 1161 (1893–6543)
Isocenter to tumor distance (cm)	5.6 ± 1.9 (3.4–9.5)
Tumor location (left/right/bilateral lung)	(5/3/6 patients)

### 2.B Clinical 6X‐FFF plans and treatment delivery

A single isocenter was placed approximately equidistant to the separate tumors in each patient. Average isocenter to tumor distance was 5.6 ± 1.9 cm (ranged 3.4–9.5 cm). Highly conformal, clinically optimal VMAT treatment plans were generated on the free‐breathing 3D‐CT scan using 2–6 co/non‐coplanar full/partial arcs (5–10°, couch kicks were used for non‐coplanar partial arcs) for the Truebeam linear accelerator (Varian, Palo Alto, CA) with millennium MLC and a 6 MV‐FFF (1400 MU/min) beam. All clinical plans were optimized in Eclipse TPS (version 13.6) with photon optimizer algorithm using a fixed 2.5 mm voxel resolution. For each arc, collimator angles were chosen manually such that the opening of the MLC between/among the tumors was minimized for each patient. Additionally, the jaw tracking option was applied during plan optimization to further minimize the nontarget OAR dose. Advanced Acuros‐based dose calculation algorithm^29^, ^30^, ^31^, ^32^ and dose to medium dose reporting option was used. A dose of 54 or 50 Gy in three and five fractions was prescribed to the 70–80% isodose line with at least 95% of the each PTV receiving the prescription dose. Optimization constraints were used to ensure a hot spot between 120–140% of the prescription dose fell within the center of the PTV. Optimization ring structures of 0.5 cm were created 2 cm away from the PTV and optimized to receive a dose <50% the prescription dose using a priority between 90 and 100. In addition to optimization ring structures, manual normal tissue objective (NTO) parameters were used to control the gradients for each target. Normal tissue objective with a high priority of 150, distance to target border of 0.1 cm, start dose of 100%, and end dose of 40% were used with a fall‐off factor of 0.4 mm^−1^. Planning objectives for the OAR were per RTOG 0915 guidelines.^4^

For the clinical 6X‐FFF VMAT plans, planning and delivery dose agreement was assessed with an Octavius phantom (PTW, Freiburg, Germany). For Octavius quality assurance (QA) plans, the average pass rates for the single‐isocenter/multiple‐lesion VMAT lung SBRT plan were 98.8 ± 2.5% for 3%/3 mm clinical gamma pass rate criteria and the maximum point dose measurement was 1.0 ± 0.7%. The beam‐on time was calculated by using dose rate of 1400 MU/min for these plans. The delivered dose rate was confirmed by reviewing each VMAT arc (control points) for all patients under the MLC properties in Eclipse TPS. Additionally, maximum dose rate of 1400 MU/min was visually observed during VMAT QA delivery on Truebeam for all single‐isocenter/multiple‐lesion lung SBRT plans.

Before delivering each 6X‐FFF VMAT lung SBRT treatment, a daily QA check on kilovoltage to megavoltage imaging isocenter coincidence was performed, including IsoCal tests for precise and accurate target localization. The daily IsoCal localization accuracy for Truebeam was <0.5 mm. All the QA procedures complied for SBRT treatment delivery. The patients were set up using daily cone‐beam CT scans following an image‐guidance SBRT procedure established in our department. Patients were treated every other day.

### 2.C Re‐optimized 6X‐FF plans

For comparison, the 6X‐FFF VMAT plans for all SBRT patients were retrospectively re‐optimized with 6X‐FF beam (600 MU/min). Identical beam geometry, planning objectives, and convergence mode were used in the 6X‐FFF and 6X‐FF plans, including the normal tissue objectives (NTO) parameters and ring structures. The 6X‐FF plans received the same target coverage as the clinical 6X‐FFF plans.

### 2.D Re‐optimized 10X‐FFF plans

Furthermore, the 6X‐FFF VMAT plans for all SBRT patients were retrospectively re‐optimized with 10X‐FFF beam (2400 MU/min). Identical beam geometry, planning objectives, and convergence mode were used in the 6X‐FFF and 10X‐FFF plans including the NTO parameters and ring structures as described above. The 10X‐FFF plans received the same target coverage as the clinical 6X‐FFF plans.

### 2.E Plan comparison

The dose volume histograms (DVHs) and isodose curves of 6X‐FFF, 6X‐FF, and 10X‐FFF plans were compared. Conformity index (CI), heterogeneity index (HI), gradient index (GI), gradient distance (GD), and maximum dose to 2 cm away in any direction from the PTV (D2cm[%]) were calculated per RTOG 0915 recommendation. The dose to the normal lung was evaluated using V5Gy[%], V10Gy[%]_y_, V20Gy[%], mean lung dose (MLD), and maximum dose to 1000 cc of lungs (D1000cc[Gy]). Dosimetric disparities were evaluated for spinal cord, heart, bronchial tree, esophagus, ribs, and skin per RTOG guidelines. Total number of monitor units (MU), modulation factor (MF), and beam‐on time were assessed. The MF is defined as the total number of MU divided by the prescription dose in cGy and the corresponding beam‐on time was calculated using total MU divided by the delivered dose rate for each beam type. Statistical analysis was performed using Microsoft Excel (Microsoft Corp., Redmond, WA). Paired sample t‐tests were used to compare differences with *P* < 0.05 considered statistically significant.

### 2.F Clinical follow‐up

Clinical outcomes evaluated include tumor local‐control rates, radiation pneumonitis, and rib toxicity. Kaplan‐Meier (KM) estimates of time‐to‐local failure were investigated (XLSTAT, Microsoft Excel). Patient follow up included physical exam followed by CT scan every 3 months for the first year and then as clinically indicated.

## 3 RESULTS

### 3.A Treatment plan and delivery parameters

**Fig. 1:**
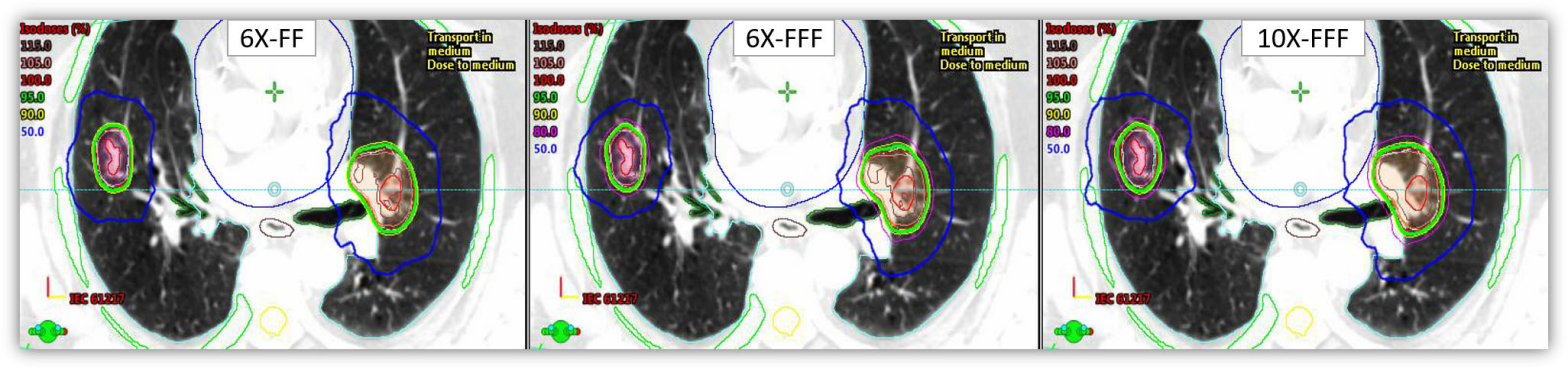
Isodose distribution obtained through the central axis for 6X‐FF, 6X‐FFF, and 10X‐FFF plans. This patient had two lesions, each treated to 50 Gy in five fractions. 6X‐FFF (center panel) showed improved target coverage and lower dose to the organs at risks compared to 6X‐FF and 10X‐FFF beams. Combined ITV (red), combined PTV (purple), spinal cord (yellow), heart (blue), normal lung (sky blue), bronchial tree (green), and ribs (green) are shown.

**Fig. 2:**
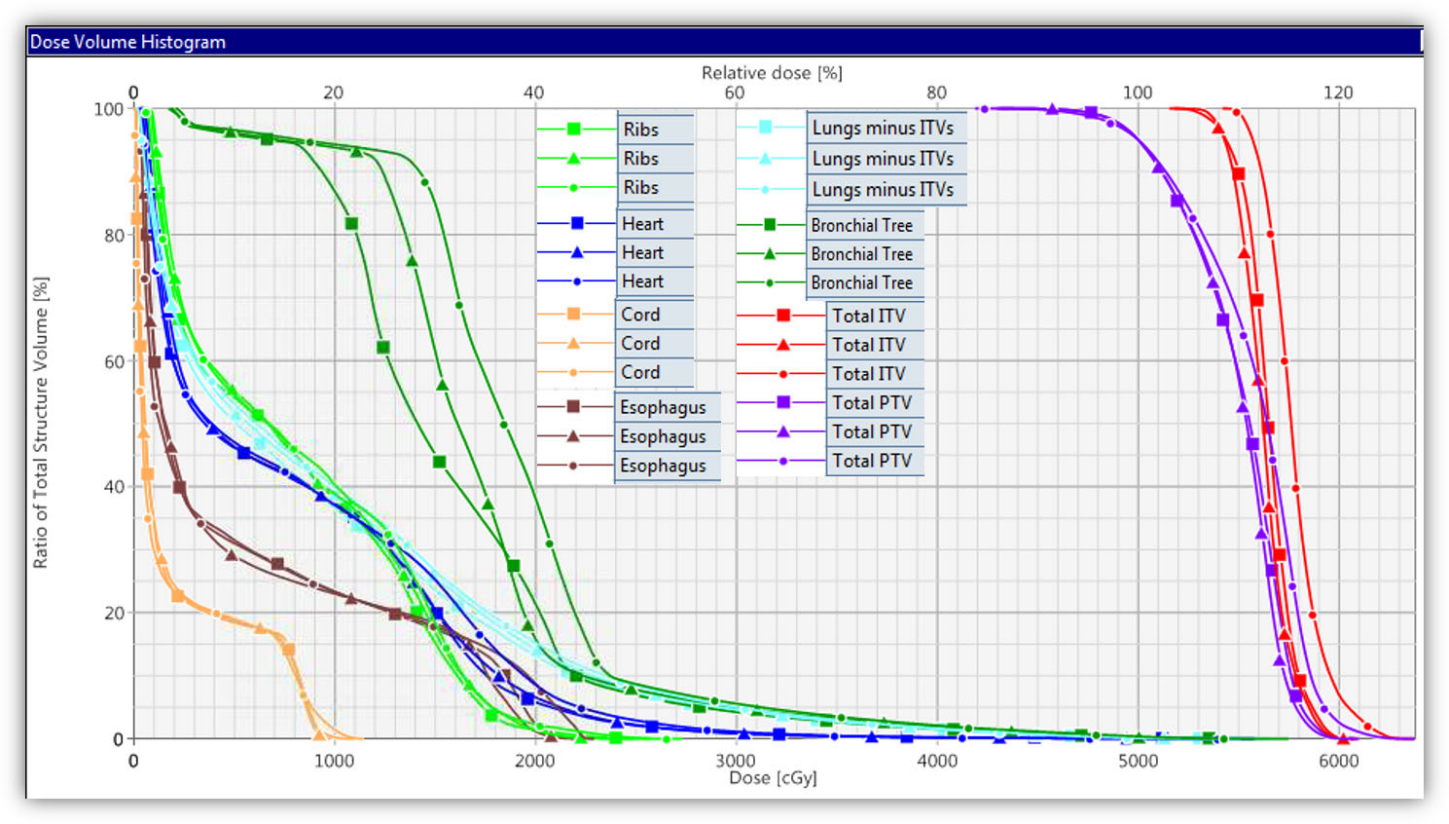
An representative dose volume histogram comparison of target coverage for combined PTV (purple), combined ITV (red), and a few organs at risks (OARs) such as total normal lung (light blue), heart (dark blue), ribs (green), esophagus (brown), bronchial tree (dark green), and spinal cord (orange) are shown for the same patient presented in Fig. 1. Triangle (6X‐FF), square (6X‐FFF), and circle (10X‐FFF). Prescription was 50 Gy in five fractions for both lesions. In this case, 10X‐FFF gave a slightly higher tumor dose to ITV, but at the cost of relatively higher doses to the other OARs including rib and bronchial tree. However, skin dose was slightly lower.

All plans were acceptable per RTOG criteria; however, statistically significant differences were observed in dose distributions and corresponding dose volume histograms when comparing the three VMAT plans (6X‐FF, 6X‐FFF, and 10X‐FFF) for the same patient, as shown in Fig. 1. The corresponding dose volume histogram for the same patient is shown in Fig. 2. Obtained dose distributions were more conformal with 6X‐FFF plans (average CI < 1.05) and exhibited steep dose fall‐off outside the combined PTV (average GI < 5.5; GD < 1.5 cm; and D2cm < 50.1%) compared to 6X‐FF (average CI < 1.08; GI < 5.8; GD < 1.6 cm; and D2cm < 52.0%) and 10X‐FFF plans (average CI < 1.17; GI < 6.2; GD < 1.66 cm; and D2cm < 53.7%), respectively. 6X‐FFF plans exhibited improved combined PTV conformity and reduced intermediate dose spill outside the target (s). Due to the dose reduction in the surrounding lung tissue, the V20Gy[%] was reduced by 7% and 17% on average, respectively, compared to 6X‐FF and 10X‐FFF beams. A detailed summary of the dosimetry and treatment delivery data for each beam type is provided in Table 2.

**Table 2:** Evaluation of plan quality for 6X‐FF, 6X‐FFF, and 10X‐FFF plans for all lung stereotactic body radiotherapy (SBRT) patients treated with single‐isocenter/multiple‐lesions VMAT lung SBRT. Prescription was 54 Gy (n = 7)/50 Gy (n = 7) in 3/5 fractions. Mean ± SD (*P*‐value) was reported. Modulation factor (MF) = total MU/prescription (cGy). n.s. = not significant. Significant values were highlighted in bold.

Target & OAR	Parameters	6X‐FF	6X‐FFF	10X‐FFF	6X‐FF vs. 6X‐FFF	6X‐FF vs. 10X‐FFF	6X‐FFF vs. 10X‐FFF
Combined PTV	CI	1.08 ± 0.01	1.05 ± 0.01	1.13 ± 0.01	*n. s*.	*n. s*.	***P = 0.04***
	HI	1.24 ± 0.01	1.22 ± 0.01	1.26 ± 0.01	***P < 0.001***	***P = 0.001***	***P < 0.001***
	GI	5.8 ± 2.1	5.5 ± 2.1	6.2 ± 2.3	***P = 0.003***	***P = 0.001***	***P < 0.001***
	D2cm[%]	51.9 ± 27.3	50.1 ± 27.5	53.7 ± 50.1	***P < 0.001***	***P < 0.001***	***P < 0.001***
	GD [cm]	1.57 ± 0.1	1.49 ± 0.1	1.66 ± 0.1	***P < 0.001***	***P < 0.001***	***P < 0.001***
Maximal dose to OAR	V20Gy[%]	6.9 ± 3.4	6.4 ± 3.1	7.7 ± 3.7	***P < 0.001***	***P < 0.001***	***P < 0.001***
	Skin [Gy]	17.0 ± 4.0	16.9 ± 4.0	15.9 ± 4.0	*n. s*.	***P = 0.04***	***P = 0.04***
	Ribs [Gy]	40.8 ± 11.9	40.1 ± 13.3	42.5 ± 12.2	*n. s*.	*n. s*.	***P = 0.01***
	Spinal cord [Gy]	11.0 ± 3.6	10.5 ± 3.6	12.0 ± 4.3	*n. s*.	***P = 0.004***	***P < 0.001***
	Heart [Gy]	24.1 ± 11.5	23.5 ± 11.3	25.4 ± 11.5	*n. s*.	***P = 0.02***	***P = 0.001***
	Bronchial tree [Gy]	24.2 ± 12.9	23.4 ± 13.2	25.3 ± 13.6	*n. s*.	***P = 0.003***	***P < 0.001***
	Esophagus [Gy]	18.4 ± 7.6	17.5 ± 7.4	19.6 ± 8.1	***P = 0.03***	***P = 0.01***	***P < 0.001***
	Total # of MU	5161 ± 2257	4640 ± 1786	5282 ± 2363	***P < 0.001***	*n. s*.	***P < 0.001***
	MF	3.7 ± 0.7	3.3 ± 0.7	3.8 ± 1.0	***P < 0.001***	*n. s*.	***P < 0.001***
	Beam‐on time [min]	8.6 ± 3.8	3.4 ± 1.38	3.0 ± 1.41	***P < 0.001***	***P < 0.001***	***P < 0.001***

Additionally, OAR sparing (ribs, spinal cord, heart, bronchus, and esophagus) was improved with 6X‐FFF beam compared to other two beams (see Table 2), except for skin, which was slightly better with 10X‐FFF beams. The absolute dose differences in the OAR were up to 1.0 Gy with all three plans and not expected to be clinically significant. Moreover, dose to 0.35 cc of spinal cord (D0.35cc[Gy]), 15 cc of heart (D15cc[Gy]), 5 cc of esophagus (D5cc[Gy]), 4 cc of bronchial tree and trachea (D4cc[Gy]), 1 cc of ribs (D1cc[Gy]), and dose to 10 cc of skin (D10cc[Gy]) met SBRT protocol requirement with all three plans. Normal lung V10Gy[%], V5Gy[%], MLD, and maximal dose to 1000 cc of lung (D1000cc[Gy]) were also slightly lower with 6X‐FFF plans (not shown here).

**Fig. 3:**
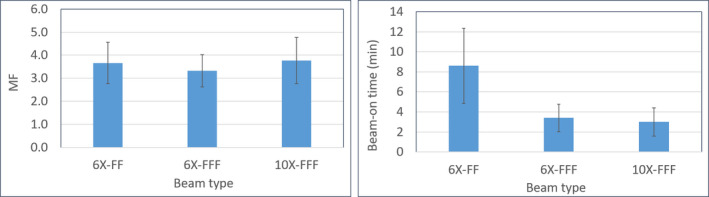
Left panel shows the average modulation factor of all 14 patients treated with single‐isocenter/multiple‐lesion volumetric modulated arc therapy lung stereotactic body radiotheraphy (SBRT) for all three plans. In the right panel, the corresponding beam‐on time is shown.

Compared to 6X‐FF and 10X‐FFF plans, 6X‐FFF plans show less beam modulation that was due to reduction in total number of MU (see Table 2). Mean MF was 3.3 for 6X‐FFF, 3.7 for 6X‐FF, and 3.8 for 10X‐FFF plans. Delivery of 6X‐FFF and 10X‐FFF plans significantly reduced beam‐on time compared to 6X‐FF plans. Mean beam‐on time was 3.4 ± 1.38 min for 6X‐FFF and 3.0 ± 1.41 min for 10X‐FFF and 8.6 ± 3.8 min for 6X‐FF plans. The average dose rate was 1400 and 1800 MU/min for 6X‐FFF and 10X‐FFF plans; while 600 MU/min was used for 6X‐FF plans. Although the 10X‐FFF has a maximum dose rate of 2400 MU/min, an analysis of the MLC control points show that the dose rate rarely reaches the maximum dose rate and has an average dose rate of 1800 MU/min (for these high dose per fraction treatments). The MF and the beam‐on time for all three plans are shown in Fig. 3. Because of reduced beam modulation and consistently achieving the maximum dose rate of 1400 MU/min, the average beam‐on time for 6X‐FFF plan was very similar to that of 10X‐FFF plans (see Fig. 3).

### 3.B Clinical follow‐up outcomes

Patient follow up included physical exam followed by CT scan every 3 months for the first year and then as clinically indicated. One patient died (unknown reason) before clinical follow up and one was lost to follow up. Of 12 patients, 11 achieved completed response to the SBRT treatment, with one lesion of one patient progressing with no reported treatment‐related lung or rib toxicity. Median follow‐up time was 9 ± 5 months (range, 3–15 months), followed up on CT scans. KM estimated 1‐yr actuarial tumor local control rate was 100%; however, 50% of the patients developed distant metastases. Table 3 shows the tumor local‐control rates and toxicity profiles on a per‐patient basis. During the follow‐up period, no patient had adverse pulmonary effects, developed grade+2 pneumonitis, or had chest wall pain/rib fracture. However, four patients developed radiographic changes of evolving lung fibrosis (grade 1 pneumonitis) that were asymptomatic in nature.

**Table 3:** Treatment outcome (n = 12). Total delivered dose: 54 Gy/50 Gy to each lesion in 3/5 fractions.

Patient	Total delivered dose (Gy) to each lesion (tumor location)	Local control (months)	Recurrence	Lung toxicity	Ribs toxicity
			Local/distant Time to progress	Acute/grade Late/grade	Acute/grade Late/grade
1	54 (bilateral lungs)	3, both lesions control	None	None	None
2	54 (bilateral lungs)	15, both lesions control	None	Grade 1	None
3	50 (bilateral lungs)	12, both lesions control	None	Grade 1	None
4	54 (Lt lung)	15, both lesions control	Brain distant, 15 months	Grade 1	None
5	54 (bilateral lungs)	15, RUL control LLL increasing	Rt adrenal distant, 15 months	None	None
6	54 (Rt lung)	4, both lesions control	None	None	None
7	50 (bilateral lungs)	6, both lesions control	None	None	None
8	50 (Lt lung)	8, both lesions control	New lesion RUL, 6 months	None	None
9	50 (Rt lung)	3, both lesions control	Liver distant, 8 months	None	None
10	54 (bilateral lungs)	6, both lesions control	None	None	None
11^a^	54 (Lt lung)	6, all 3 lesions control	Rt lung distant, 4 months	None	None
12^b^	50 (Rt lung)	6, all 5 lesions control	New lesions on RLL, 6 months	Grade 1	None

Rt lung = right lung, Lt lung = left lung.

^a^ Note that patient #11 had three lung lesions in the left lung.

^b^ Patient #12 had total five lesions in the right lung. For all patients, all lung lesions were treated synchronously using a single‐isocenter VMAT SBRT plan.

## 4 DISCUSSION

Implementation of 6X‐FFF beam for stereotactic treatment of synchronous multiple lung lesions using a single‐isocenter VMAT plan at a maximum dose rate (1400 MU/min) is clinically feasible and advantageous. In this series, all patients had multiple metastatic lung lesions (2–5 lesions). Furthermore, many of these patients were elderly with associated comorbidities and unable to retain their treatment positions adequately for an extended period. Single‐isocenter VMAT plans with 6X‐FFF beam produced clinically optimal dose distributions and, at high‐dose rates, substantially shorted the beam‐on time and improved treatment feasibility. 6X‐FFF provided highly conformal target coverage, lower intermediate dose spillage, and statistically significant OAR sparing compared to 6X‐FF and 10X‐FFF plans, as demonstrated in Table 2. However, the absolute dose differences were not clinically significant and doses with each beam were well below RTOG‐required tolerances for all plans with one exception (rib dose was higher with 10X‐FFF, up to 2.0 Gy higher compared to 6X‐FFF plans). Skin sparing was slightly better with 10X‐FFF plans. Furthermore, 6X‐FFF plans provided lower maximal doses to all OAR (spinal cord, heart, esophagus, ribs, and skin). This includes less normal lung V20Gy[%], which is reduced due to the shorter range of secondary electrons produced by the 6X‐FFF beam. The 6X‐FFF plans required less MU to deliver the same prescription dose due to less beam modulation across the target, minimizing beam‐on time (see Table 3). This could be partly due to the quick dose buildup at tumor–lung interface with 6X‐FFF beam (characteristic of 6X‐FFF beam), there were less total number of MU required (for same prescribed dose) compared to 6X‐FF and 10X‐FFF beams, potentially reducing the beam modulation and providing smaller MF. Less beam modulation is desirable (less dose leakage) in the treatment of multifocal lesions with a single‐isocenter VMAT plan since MLCs must travel longer distances between the tumors.

Limited data are available describing lung SBRT using FFF‐VMAT.^13^, ^14^, ^15^ Navarria and colleagues reported on patients treated with SBRT for medically inoperable early‐stage NSCLC patients. Eighty‐six patients received the traditional flattened beam with 3D‐conformal plans and 46 patients received VMAT treatment with 6X‐FFF beam. All patients were treated with 48 Gy in four fractions. Interestingly, they observed earlier tumor responses in the 6X‐FFF group and 1‐yr local control rate of 100% vs. 92.5% in the 6X‐FF group (*P* = 0.03). No difference in pulmonary toxicity was observed. Rieber et al.^15^ reported early results of 61 pulmonary lesions treated with SBRT using FFF beam. They reported a low 5% rate of early grade ≥ 2 toxicity and a 1‐yr tumor local‐control rate of 92.8%. These data were for treating a single lung lesion with an isocenter at the center of the tumor. In contrast to these studies, patients in the current series were treated using a single‐isocenter VMAT plan with 6X‐FFF beam to multiple lung lesions, synchronously. The single‐isocenter VMAT planning technique can deliver fast (average beam‐on time 4.3 min) and effective treatment. Our study demonstrated superior plan quality (with 6X‐FFF beam) with the lowest dose to OAR and promising early clinical outcomes of estimated 1‐yr actuarial tumor local control rate of 100% and no incidence of treatment‐related acute side‐effects. Although six patients had distant failure, the local control of treated lesions could preserve the patient’s quality of life or delay chemotherapy treatment. Future research includes long‐term clinical follow up to determine tumor local control rates and late treatment‐related toxicities.

A potential concern for SBRT treatment of lung lesions is dose spill in the chest wall and ribs,^33^, ^34^, ^35^ normal lung (V20Gy[%], V10Gy[%], and V5Gy[%]),^36^, ^37^, ^38^ and acute skin toxicity.^39^ Pettersson et al.^33^reported 68 NSCLC patients treated to 45 Gy in three fractions with lung SBRT. Among the 33 patients with complete clinical and radiographic follow up exceeding 15 months, 13 ribs fractures were found in seven patients. The logistic dose–response curve showed that the risk of radiation‐induced rib fracture was related to dose to 2 cc of the rib. With a median follow up of 29 months, they showed that 2 cc of rib receiving 27.3 Gy (3 × 9.1 Gy) had 5% probability of rib fracture. In our current study, utilizing 6X‐FFF beam for VMAT treatment of synchronous lung lesions produced dose metrics for rib and other OAR that were improved compared to other 6X‐FF and 10X‐FFF plans. Therefore, there was no acute toxicity and late toxicities are expected to occur at a lower rate.

Another concern is the interplay effect of a change in breathing patterns with MLC modulation, gantry rotation, and dose‐rate changes during dose delivery that is not likely to average out during relatively short beam‐on times. It has been reported that the interplay effect causes insignificant dose blurring when using two or more arcs, in agreement with this study.^40^ The change in respiratory patterns between CT simulation and time of treatment has been studied previously and only small changes (within ±3 mm) due to intrafractional and interfractional motion.^41^, ^42^ A symmetric 5 mm PTV margin around the ITV was recommended to address these potential motion errors. Although there is little guidance concerning PTV margin in the case of a single‐isocenter/multilesion SBRT treatment, the recommendation of this study is to use abdominal compression for these cases. Further expansion of this margin would deliver unwanted dose to normal lung thus a 5 mm margin should be maintained until further research on this topic is completed. In this study, the use of multiple 6X‐FFF partial‐arcs and co/non‐coplanar VMAT planning with variable collimator rotations yielded an average beam‐on time of 3.4 min, potentially decreasing the variation of intrafraction motion error and improving the treatment accuracy and efficiency. Compared to traditional 6X‐FF plans, overall reduction in beam‐on time of 65% and 60% was achieved when using 10X‐FFF and 6X‐FFF plans, respectively. Additionally, a 12% (0.4 min) reduction in the average beam‐on time was found with 10X‐FFF plans compared to 6X‐FFF plans, but at the cost of a less conformal plan and higher dose to OAR, including rib. Furthermore, due to the larger number of total MU delivered in the SBRT treatment of lung lesions, photonuclear production could be a concern with 10X‐FFF beam, although neutron production has been shown to be minimal provided that the treatment plans for each beam required approximately the same number of total MU, yet it was found that higher photoneutron yield per source electron still existent with 10X‐FFF beam.^43^ Because of this dosimetric study, 6X‐FFF plan was the choice in our clinic for lung SBRT treatment with smaller number of total MU, higher plan quality, and lower dose to OAR, including the treatment of synchronous multifocal lung lesions (see Table 2). Moreover, our early clinical follow‐up results (mean, 9 months) are encouraging with 100% local tumor‐control rate and no reported treatment‐related acute toxicity.

## 5 CONCLUSION

6X‐FFF beam for SBRT treatment of multiple lung lesions using a single‐isocenter VMAT plan yielded superior plan quality and faster treatment delivery compared to conventional 6X‐FF beam — perhaps improving clinic efficiency and patient comfort. It has been observed that utilizing 10X‐FFF beam could marginally improve the delivery efficiency and skin dose (only for selected patients) but at the cost of inferior dose conformity, gradient indices (higher intermediate dose spill), and higher dose to other OAR. Thus, 6X‐FFF beam was chosen in our clinic for lung SBRT patients including synchronous treatment of multiple lung lesions via a single‐isocenter VMAT plan. The single‐isocenter VMAT technique was fast, effective, and well tolerated by all patients, improving patient compliance and potentially reducing the amount of intrafraction motion errors for well‐suited patients. Our early clinical outcomes are promising in terms of local control rates with no acute side effects to lung or ribs and potentially improving the patient’s quality of life. Longer clinical follow up of these patients is underway.

## CONFLICT OF INTEREST

None.
